# Double-level knee valgization osteotomy has high survivorship and a low complication rate in a single-center series of 58 cases with a mean clinical follow-up of 10 years

**DOI:** 10.1186/s43019-025-00271-8

**Published:** 2025-05-12

**Authors:** Ahmed Mabrouk, Michael Risebury, Sam Yasen

**Affiliations:** Department of Trauma and Orthopaedics, Basingstoke and North Hampshire Hospital, Basingstoke, England, UK

**Keywords:** Double level knee osteotomy, Valgization osteotomy, Varus Knees, Survival, Outcomes

## Abstract

**Background:**

Double-level knee osteotomy (DLO) is becoming more popular in bifocal (femur and tibia deformities) as it addresses the deformity where it belongs and results in a more physiologic joint line obliquity. This study reports on the early to midterm outcomes, both clinical and radiological, of valgization DLO for varus knees and the first study to report the 10-year survivorship of this procedure.

**Methods:**

A retrospective review of a prospectively maintained single-center database of 1170 knee osteotomies was undertaken. Patients with bifocal (femur and tibia) varus malalignment and isolated medial compartment osteoarthritis who had DLO corrections [high tibial osteotomy (HTO) and distal femoral osteotomy (DFO)] were included. Multiple patient-reported outcome measures (PROMs) were recorded preoperatively and serially postoperatively. This included the Knee Injury and Osteoarthritis Outcome Score, the Oxford Knee Score, the Oxford Knee Score—Activity and Participation Questionnaire, the Western Ontario and McMaster University Score, the visual analog scale for health and pain, and the EQ-5D. EQ-5D stands for *EuroQol 5-dimension*. It is a standardized instrument for measuring health-related quality of life (HRQoL). All lower limb alignment indices were recorded pre-and postoperatively. The rates of osteotomy revision, conversion to arthroplasty, complications, and both 8- and 10-year survivorship were recorded.

**Results:**

A total of 58 valgization DLO cases were followed up to a mean of 10.8 ± 3 years. This comprised 74.1% males and 25.9% females, with a mean age of 47.9 ± 9.8 years and a mean body mass index (BMI) of 31.5 ± 6.3 kg/m^2^. The mean planned correction angles for HTO and DFO were 7.7° ± 2.7° and 7.7° ± 3°, respectively. Postoperatively, the mean mechanical tibiofemoral angle improved from −12.7° ± 3.9° (varus) to −0.4° ± 3.4° (i.e., centered around mechanical neutral), the mean medial proximal tibial angle improved from preoperative 84.3 ± 3.2° to postoperative 90° ± 2.5°, the mean mechanical lateral distal femoral angle improved from preoperative 91.6° ± 3.4° to postoperative 86.7° ± 2.5°, and the mean Mikulicz point improved from −5 ± 13.4% to 47.7 ± 14.7% (all *p*-values < 0.001). All PROMs significantly improved at 24 months follow-up (all *p* values < 0.001). The rate of osteotomy revision was 3.4%. The overall rate of total knee arthroplasty conversion was 5.2% at an average of 5.9 ± 3.1 years postoperatively. The complication rate was 8.6%. The 8- and 10-year survivorship was 97.1%, and 94.4%, respectively.

**Conclusions:**

In this single-center series evaluating patients with varus knees and bifocal deformities, valgization double-level knee osteotomy (DLO) demonstrated favorable clinical outcomes, accompanied by a low complication rate of 8.6% and a 10-year survivorship of 94.4%. Radiographic findings from available imaging data were positive, although long-term imaging was not consistently obtained.

*Level of evidence* IV retrospective cohort study.

## Background

There has been a swift evolution of osteotomies around the knee over the past three decades [1]. This is even more pronounced over the last decade, with a far better understanding of the indications, preoperative planning, surgical strategies, complications and their management [2–6]. In addition, the advent of angular stable fixation devices and the availability of a variety of void fillers, has increased confidence in varying the osteotomy techniques and pushing the correction limits [7–11].

Valgization re-alignment, with medial opening wedge high tibial osteotomy (MOWHTO) for varus knees, has demonstrated improved clinical outcomes, low complications, and high survivorship [12–15]. Nevertheless, valgization re-alignment with a lateral closing wedge distal femoral osteotomy is indicated when the varus deformity is located at the femur to avoid creating a secondary deformity [16]. Therefore, correcting varus malalignment only at the tibia, in the absence of tibial varus or the presence of concomitant femoral varus, can result in excessive joint line obliquity (JLO) [17]. An increased JLO of 5° or more can induce excessive shear stresses on the articular cartilage, which is detrimental to knee preservation [18]. Hence, in cases of bifocal varus deformities, valgization double-level osteotomy (DLO) of both the distal femur and proximal tibia should be employed [2].

In recent years, there have been several reports on the promising short term outcomes following DLO for varus knees [19–32]. In addition, a significant increase in postoperative joint line obliquity and medial proximal tibial angle was consistently reported following MOWHTO compared to DLO [19, 33]; whereas, discrepancies in postoperative clinical outcomes, following either procedure, remain controversial. Despite no significant changes reported in some patient reported outcome measures, such as the Knee Injury and Osteoarthritis Outcome Score (KOOS) and the Knee Society Score (KSS ), other scores were reported to be higher following DLO compared to MOWHTO, such as Lysholm scores [19] and UCLA (University of California, Los Angeles) scores [33].

While valgization DLO has been shown to achieve improved coronal alignment and short term outcomes, long-term survivorship and functional outcomes remain scarcely documented compared with single-level osteotomy techniques [34]. For this reason, we aimed to present a single-center long-term analysis of survivorship and intermediate-term analysis of functional outcomes for 58 cases of valgization DLO performed for varus malaligned knees. It was hypothesized that valgization DLO results in improved clinical outcomes at the intermediate term, a high 10-year survivorship, and low complication rates.

## Patients and methods

**Fig. 1:**
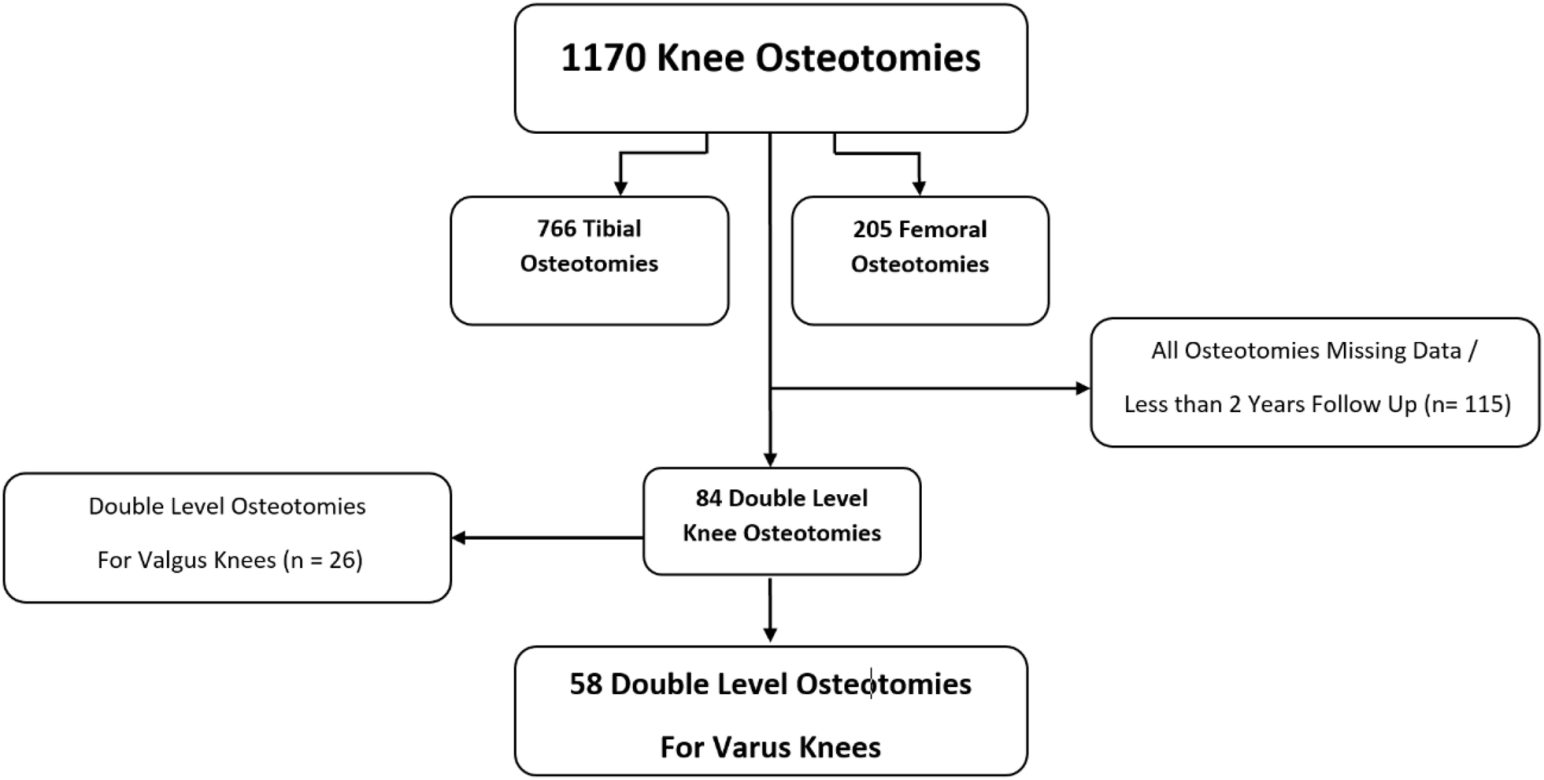
Patient flow chart

After local institutional review board approval, a retrospective analysis was conducted of a prospectively maintained single-center osteotomy database including 1170 cases of osteotomies around the knee. Included in the study are young active patients who had double-level valgization knee osteotomies for symptomatic isolated medial knee compartment osteoarthritis (OA), Kellgren–Lawrence (KL) grade I–IV, and varus knee malalignment, who exhausted all conservative treatment modalities [35]. Valgization DLO was indicated if deformity analysis identified an extra-articular deformity in both the femur (mechanical lateral distal femoral angle [mLDFA] > 90) and the tibia (medial proximal tibial angle [MPTA] < 85), or if planning a single-level osteotomy indicated that postoperative joint line obliquity would exceed 5 ° or the postoperative MPTA would exceed 94° [2]. Excluded cases comprised 766 tibial osteotomies, 205 femoral osteotomies, 26 double-level varization osteotomies, and 115 cases with missing data or follow-up of less than 2 years. The excluded cases who had a single level osteotomy had a deformity solely based in one bone, hence DLO was not indicated (Fig. 1).

### Clinical and radiographic assessment

Patients’ demographics, smoking status, and comorbidities in the form of perioperative self-administered comorbidity questionnaire (SCQ) scores were recorded. The preoperative and postoperative clinical assessment involved examination by a clinician and an independent research physiotherapist who collected patient-reported outcome measures (PROMs) preoperatively and at 6 months, 1, 2, and 5 years postoperatively. The recorded PROMs comprised: Knee Injury and Osteoarthritis Outcome Scores (KOOS), the Oxford Knee Score (OKS), the Oxford Knee Score—Activity and Participation Questionnaire (OKS-APQ), the Western Ontario and McMaster University Score (WOMAC), the visual analog scale (VAS) for health and pain, and the EQ-5D. EQ-5D stands for *EuroQol 5-dimension*. It is a standardized instrument for measuring health-related quality of life (HRQoL). In EQ-5D analysis using the Paretian Classification of Health Change (PCHC):

“Total with problems” refers to the number of individuals who had at least one *dimension with problems* (i.e., a score of 2 or 3) at any time point.

Standard knee radiographs were performed preoperatively and postoperatively at 6 weeks, and at 3, 6, and 12 months to monitor the osteotomy union (and longer if not fully united or clinically indicated). Radiological assessments with long-leg standing (LSRs) was conducted at 3 months postoperatively routinely for every case, and repeated if clinically indicated at any time point until the latest follow-up.

On LSRs, preoperative deformity analysis and 3-month postoperative correction checks were performed as per Paley’s principles [36]. The alignment indices included the mechanical tibiofemoral angle (mTFA), the mechanical lateral proximal femoral angle (mLPFA), the mechanical lateral distal femoral angle (mLDFA), the medial proximal tibial angle (MPTA), the lateral distal tibial angle (LDTA), the joint line convergence angle (JLCA), the lower limb length (LL), and the weightbearing line (Mikulicz percentage). Correction accuracy was recorded as the deviation of the postoperatively achieved correction from the planned correction, with negative values indicating under-correction and positive values indicating over-correction.

### Preoperative planning and surgical technique

**Fig. 2:**
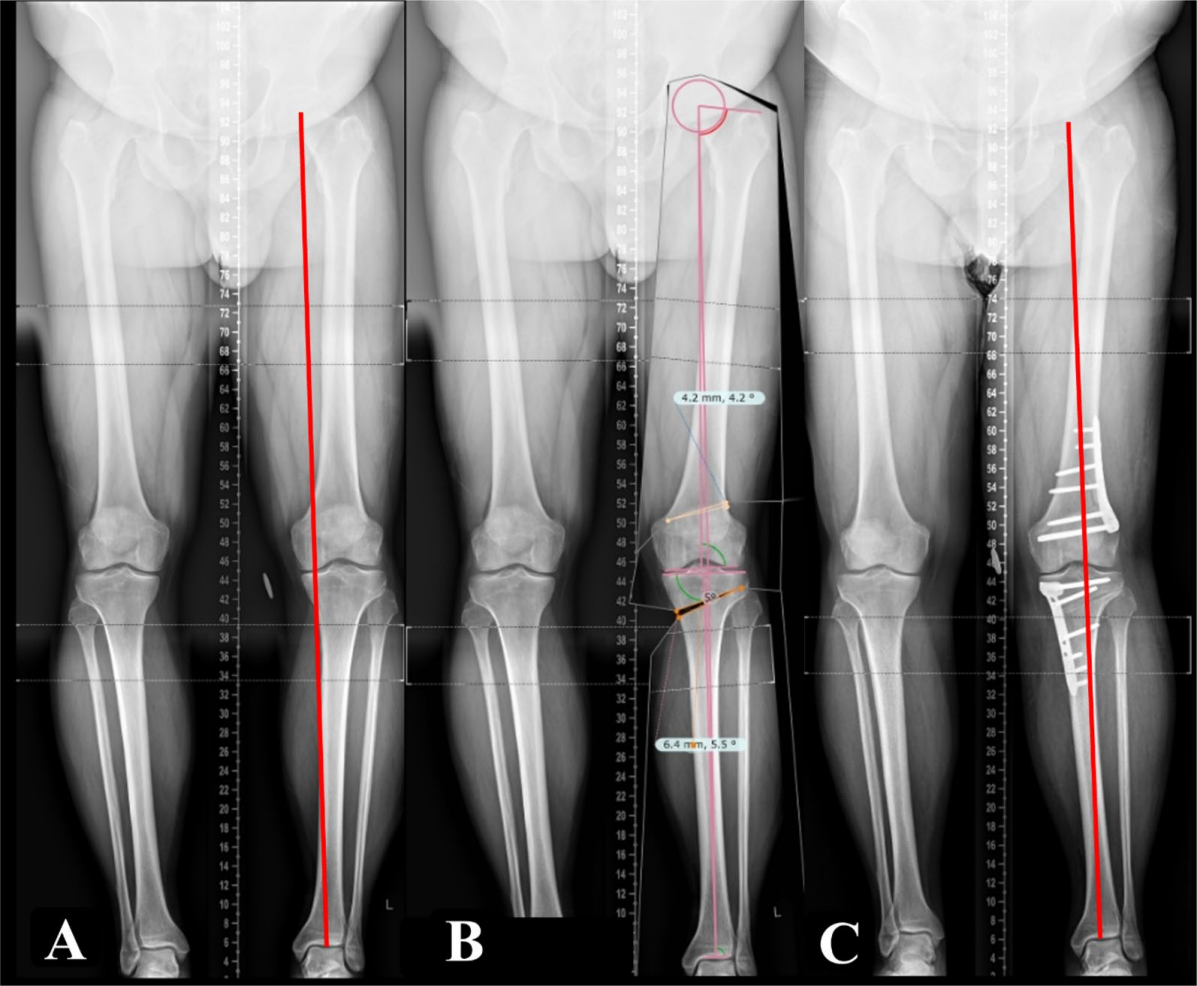
Long leg standing radiographs (LSRs). **A** Preoperative LSRs demonstrating the substantial varus malalignment of the left limb and medial deviation of the Mikulicz line. **B** Preoperative LSRs demonstrating a bespoke digitally planned correction with a MOWHTO and LCWDFO. **C** Postoperative LSRs demonstrating MOWHTO and LCWDFO, both fixed with Tomofix^®^ plates, and the corrected alignment with the Mikulicz line at 60%

Corrections were planned on digital LSRs targeting a postoperative weight-bearing axis between 50% and 60% of the tibial plateau width, measured from the medial edge. Targets around 50% were planned for cases with early OA (KL grade I) and targets around 60% were reserved for more advanced OA (KL grade IV) or early cases in the series [37] (Fig. 2). All osteotomies were performed under fluoroscopic guidance. A thigh tourniquet was used in all cases. Patients were operated on in the supine position with the knee at 90° of flexion during the surgical approach and in full extension during osteotomy execution. Tibial deformities were corrected with a proximal biplanar medial opening wedge high tibial osteotomy (MOWHTO). The osteotomy gap was either managed with a variety of void fillers, or no filler for cases earlier in the series. All tibial osteotomies were fixed with angle-stable locking plates. The detailed surgical technique of MOWHTO was previously described by the authors [14]. Measures were adopted to avoid inadvertent changes in the posterior tibial slope and patellar height as previously described in the literature [38].

**Fig. 3:**
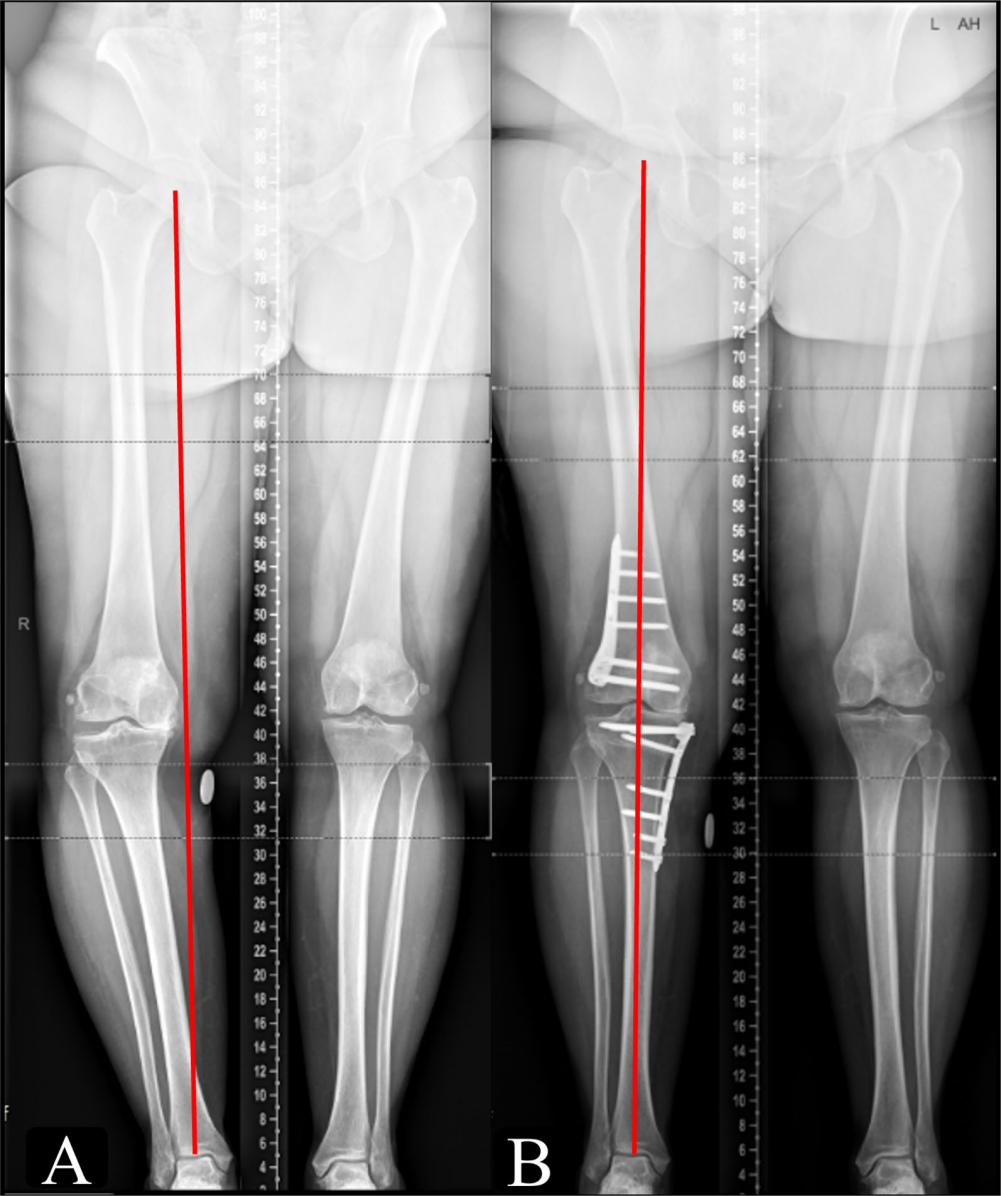
Long leg standing radiographs (LSRs) **A** Preoperative LSRs demonstrating the substantial varus malalignment of the right limb, grade IV KL isolated medial compartment osteoarthritis and significant medial deviation of the Mikulicz line. **B** Postoperative LSRs demonstrating MOWHTO and LCWDFO, both fixed with Tomofix^®^ plates, and the corrected alignment with the Mikulicz line at 50%

Femoral deformities were corrected by biplanar lateral closing wedge distal femoral osteotomy (LCWDFO). A longitudinal skin incision was made from the lateral femoral epicondyle extending proximally. A straight sharp incision was made in the iliotibial band followed by blunt dissection to identify the vastus lateralis which was retracted anteriorly. Four 2 mm Kirschner wires (K-wires), two superior and two inferior, were used to define oblique osteotomy cuts in the form of an isosceles triangle according to the calculated correction. The wires were inserted from the lateral cortex to converge at the hinge point just proximal to the superior aspect of the posterior medial femoral condyle and 5 mm from the medial cortex. The posterior wires were inserted as posteriorly as possible, and the anterior wires were inserted at approximately three-quarters of the anteroposterior thickness of the femur, allowing space for an ascending biplanar osteotomy cut. The biplanar cut was created to subtend an angle of approximately 110° from the main transverse osteotomy. The bone wedge was then extracted and the osteotomy was closed cautiously with an axial load applied through the limb. With axial pressure maintained, the osteotomy was fixed using angle-stable osteotomy locking plates (Fig. 3).

Postoperative rehabilitation in all cases involved an immediate free range of motion with no brace, partial weight-bearing with elbow crutches for 6 weeks, which progressed to weight-bearing as tolerated with crutches for a further 6 weeks, with walking aids discarded at 12 weeks. Routine thromboprophylaxis was in the form of Aspirin 75 mg daily for 6 weeks. Other measures for thromboprophylaxis were used in higher-risk cases.

### Statistical analysis

Statistical analysis was performed with statistical software [R Core Team (2024) R Foundation for Statistical Computing, Vienna, Austria]. Continuous variables that are descriptive were reported as means ± standard deviations [95% confidence intervals]. The normality of data distribution was evaluated by the Shapiro–Wilk test. Intra-group differences in lower limb alignment indices were evaluated with either the Student’s paired *t*-test or the Mann–Whitney *U* test. A linear mixed-effects model was used to evaluate changes in patient-reported outcome measures (PROMs) over time while adjusting for preoperative BMI and preoperative lower limb alignment represented by mTFA. Statistical significance was set at 0.05.

### Survival analysis

Kaplan–Meier survival analysis was employed. Conversion to arthroplasty due to symptomatic OA progression was defined as failure. Survival time was the time to conversion to arthroplasty. The length of the follow-up period was defined as the date of conversion or last observation with preserved DLO. A Cox proportional hazards model was employed to evaluate the hazard ratio (HR) of age and preoperative BMI on survivorship.

## Results

A total of 58 valgization DLO cases in 58 patients were included in the study. There were 43 males and 15 females with a mean age of 47.9 ± 9.8 years at the time of the surgery. Patients had a perioperative self-administered comorbidity questionnaire (SCQ) score of 3 ± 2.7 [95% CI 2.4, 3.5]. The mean follow-up was 10.8 ± 3 [95% CI 10.2, 11.3] years. The patient demographics are presented in Table 1. The mean planned correction angle in the HTO cases was 7.7° ± 2.7° [95% CI 6.9°, 8.5°] and in the DFO cases was 7.7° ± 3° [95% CI 6.8°, 8.6°]. Whereas, the mean planned wedge in the HTO cases was 8.3 ± 3.3 mm [95% CI 7.4 mm, 9.3 mm] and in the DFO cases was 8.1 ± 3.4 mm [95% CI 7.1 mm, 9.1 mm]. Devices used in HTO and DFO cases and void fillers are reported in Table 2.

**Table 1:** Patient demographics

Variable	Value
Age (years)	47.9 ± 9.8 [46.1, 49.7]
Gender	
Male	43 (74.1%)
Female	15 (25.9%)
BMI (kg/m^2^)	31.5 ± 6.3 [30.3, 32.7]
Smoking status	
Smoker	5 (8.6%)
Non-smoker	37 (63.8%)
Missing	16 (27.6%)
KL OA grade	
1	4 (6.9%)
2	9(15.5%)
3	13 (22.4%)
4	32 (55.2%)
Follow-up (months)	129.5 ± 35.3 [122.8, 136.1]

*BMI* body mass index, *KL* Kellgren–Lawrence, *OA* Osteoarthritis

Data reported as mean ± standard deviation [95% confidence interval]

**Table 2:** Devices used for the osteotomy fixation and the type of material used for osteotomy gap filling

	HTO (*N* = 58)	DFO (*N* = 58)
Side		
Right	27 (46.6%)	27 (46.6%)
Left	31 (53.4%)	31 (53.4%)
Device		
Tomofix LDFP (DePuy Synthes*)		54 (93.1%)
Tomofix MHT (DePuy Synthes*)	43 (74.1%)	
Tomofix MHT-SS (DePuy Synthes*)	3 (5.2%)	
Newclip ActivMotion (Newclip Technics**)	9 (15.5%)	3 (5.2%)
Arthrex PEEK Power Plate (Arthrex⁂)	1 (1.7%)	1 (1.7%)
Oatis C plate (S.B.M⁑⁑)	2 (3.4%)	
Graft		
Femoral head allograft	27 (46.7%)	
Femoral head allograft + DFO wedge	2 (4%)	
HydroxyColl^®^ (RCSI ℛ)	1 (2%)	
Precision Wedge ^®^ (RTI Surgical‡‡)	5 (9%)	
Quickset ^®^ Bone Cement (Arthrex⁂) + Precision Wedge ^®^ (RTI Surgical‡‡)	2 (4%)	
Wedge from DFO	3 (5%)	
No graft	18 (32%)	58 (100%)

^*^DePuy Synthes, Oberdorf, Switzerland; **Newclip Technics, Nantes, France; ⁂Arthrex, Naples, Florida, USA; ⁑⁑ S.B.M, Lourdes, Hautes-Pyrénées, France; RCSI ℛ, Dublin 2, Ireland; ‡‡ RTI Surgical, Marquette, Michigan, USA

**Fig. 4:**
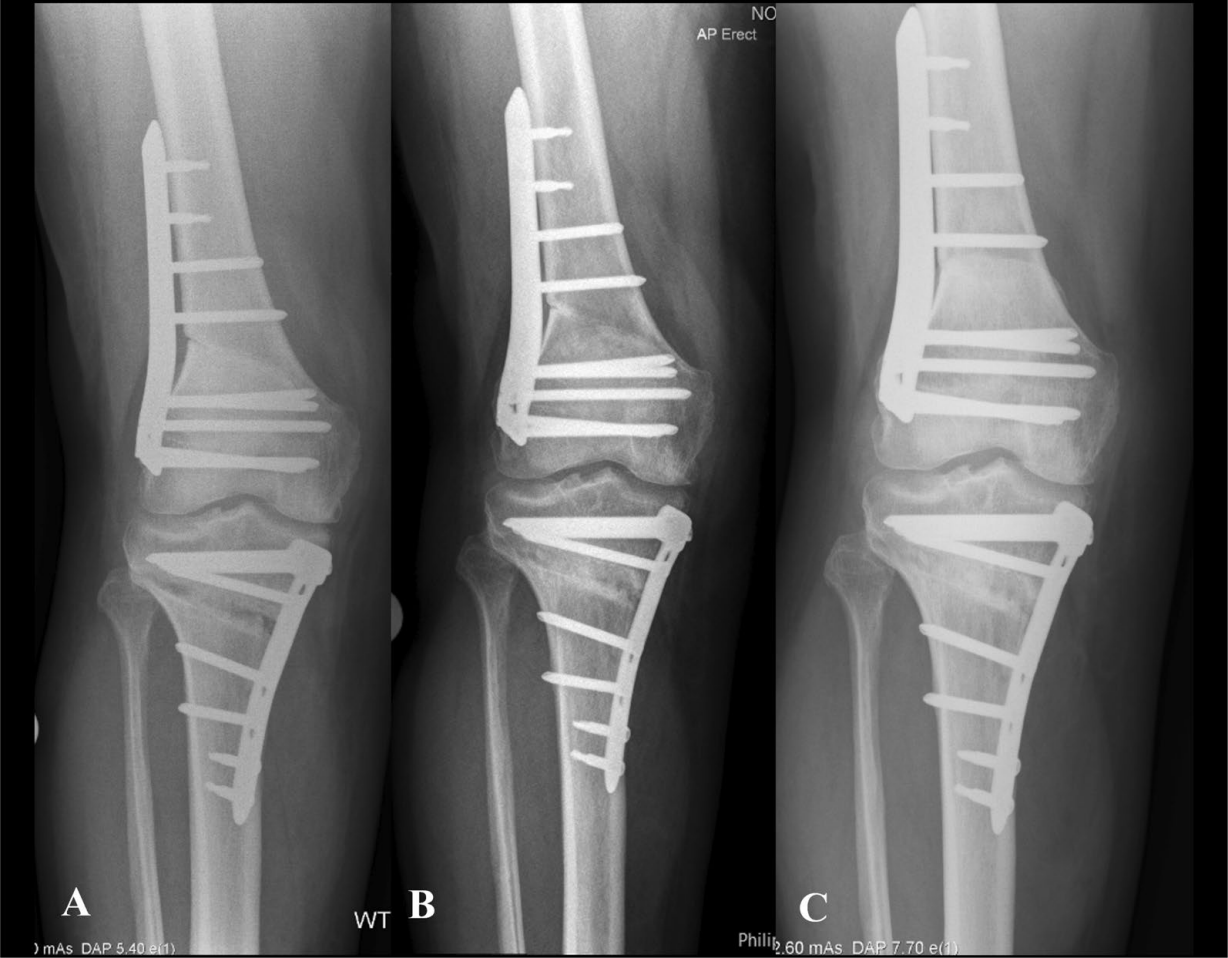
Anteroposterior weight bearing knee radiographs demonstrating progression of osteotomy union: **A** 6 weeks postoperatively; **B** 6 months postoperatively; and **C** 1 year postoperatively

**Fig. 5:**
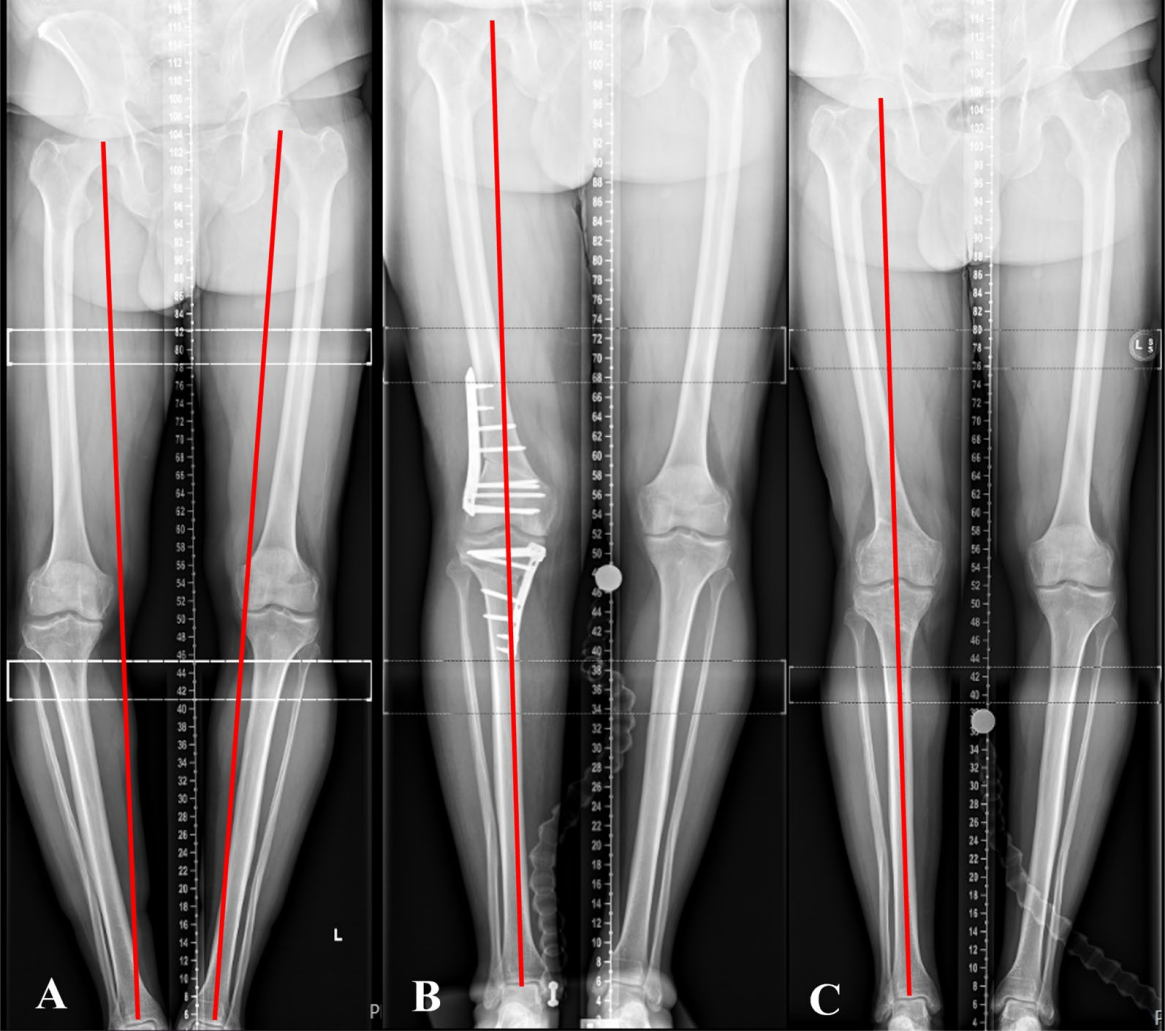
Long leg standing radiographs (LSRs). **A** Preoperative LSRs demonstrating the substantial varus malalignment of the right limb, grade III KL isolated medial compartment osteoarthritis, and significant medial deviation of the Mikulicz line. **B** Three month postoperative LSRs demonstrating MOWHTO and LCWDFO, both fixed with Tomofix^®^ plates, and the corrected alignment with the Mikulicz line at 50%. **C** Five year postoperative LSRs demonstrating the maintenance of correction of the right limb at Mikulicz 50% with both plates removed

The mean preoperative and postoperative lower limb radiographic measurements are presented in Table 3. Negative mTFA angles represent varus coronal alignment (i.e., Mikulicz under 50%) and positive values represent valgus (i.e., Mikulicz over 50%). The correction accuracy is reported in Table 4. Serial weight bearing anteroposterior knee radiographs and long leg standing radiographs are shown in Figs. 4 and 5, to demonstrate osteotomy union and maintenance of correction, respectively.

**Table 3:** Preoperative and postoperative lower limb radiographic measurements

	Preoperative values	Postoperative values	Correction values*	*p*-Values
mTFA (degrees)	−12.7 ± 3.9 [−13.4, −11.9]	−0.4 ± 3.4 [−1.1, 0.2]	12.3 ± 4.0 [11.6, 13.1]	*p* < 0.001
mLPFA (degrees)	91.9 ± 8.3 [90.3, 93.5]	90.7 ± 7.9 [89.1, 92.2]	−1.2 ± 13.7 [−5.5, −0.2]	*p* = 0.1
mLDFA (degrees)	91.6 ± 3.4 [90.9, 92.2]	86.7 ± 2.5 [86.2, 87.2]	−4.9 ± 12.7 [−9.0, −4.1]	*p* < 0.001
MPTA (degrees)	84.3 ± 3.2 [83.7, 84.9]	90 ± 2.5 [89.6, 90.5]	5.7 ± 13.0 [1.5, 6.5]	*p* < 0.001
LDTA (degrees)	87.7 ± 4.7 [86.8, 88.5]	87.2 ± 4.4 [86.3, 88]	−0.5 ± 12.4 [−4.4, 0.4]	*p* = 0.6
JLCA (degrees)	4.8 ± 2.5 [4.3, 5.3]	4.2 ± 2.0 [3.8, 4.5]	−0.6 ± 1.9 [−1.1, −0.4]	*p* = 0.03
LL (mm)	817.6 ± 70.8 [804.2, 831.0]	818.4 ± 68.6 [805.1, 831.7]	−0.8 ± 2 [−1, 1.5]	*p* = 0.8
Mikulicz (%)	−5 ± 13.4 [−7.5, −2.4]	47.7 ± 14.7 [44.8, 50.5]	52.7 ± 19.1 [48.1, 55.5]	*p* < 0.001

^*^The correction values are reported as the delta = postoperative − preoperative. All values are reported as mean ± standard deviation [95% confidence intervals]

*mTFA* mechanical tibiofemoral angle, *mLPFA* mechanical lateral proximal femoral angle, *mLDFA* mechanical lateral distal femoral angle, *MPTA* medial proximal tibial angle, *LDTA* lateral distal tibial angle, *JLCA* joint line convergence angle, *LL* lower limb length, *Mikulicz* Mikulicz line percentage

Negative mTFA is varus and positive mTFA is valgus

**Table 4:** Planned and postoperative lower limb radiographic measurements along with the correction accuracy and its significance

	Planned values	Postoperative values	Correction accuracy	*p*-Values
mTFA (degrees)	0.7 ± 1.1 [0.5, 0.9]	−0.4 ± 3.4 [−1.1, 0.2]	1.1 ± 3.5 [0.4, 1.8]	*p* = 0.01
mLDFA (degrees)	86.1 ± 2.3 [85.6, 86.5]	86.7 ± 2.5 [86.2, 87.2]	0.6 ± 2.5 [1.3, 3.4]	*p* = 0.04
MPTA (degrees)	91 ± 2.0 [90.6, 91.4]	90 ± 2.5 [89.6, 90.5]	1 ± 2.5 [0.3, 5.2]	*p* = 0.04
Mikulicz (%)	52.9 ± 4.3 [52.1, 53.7]	47.7 ± 14.7 [44.8, 50.5]	5.2 ± 15.7 [3.2, 9.2]	*p* = 0.006

^*^The correction accuracy values are reported as absolute the delta values = postoperative − planned

*mTFA* mechanical tibiofemoral angle, *mLDFA* mechanical lateral distal femoral angle, *MPTA* medial proximal tibial angle, *Mikulicz* Mikulicz line percentage

**Fig. 6:**
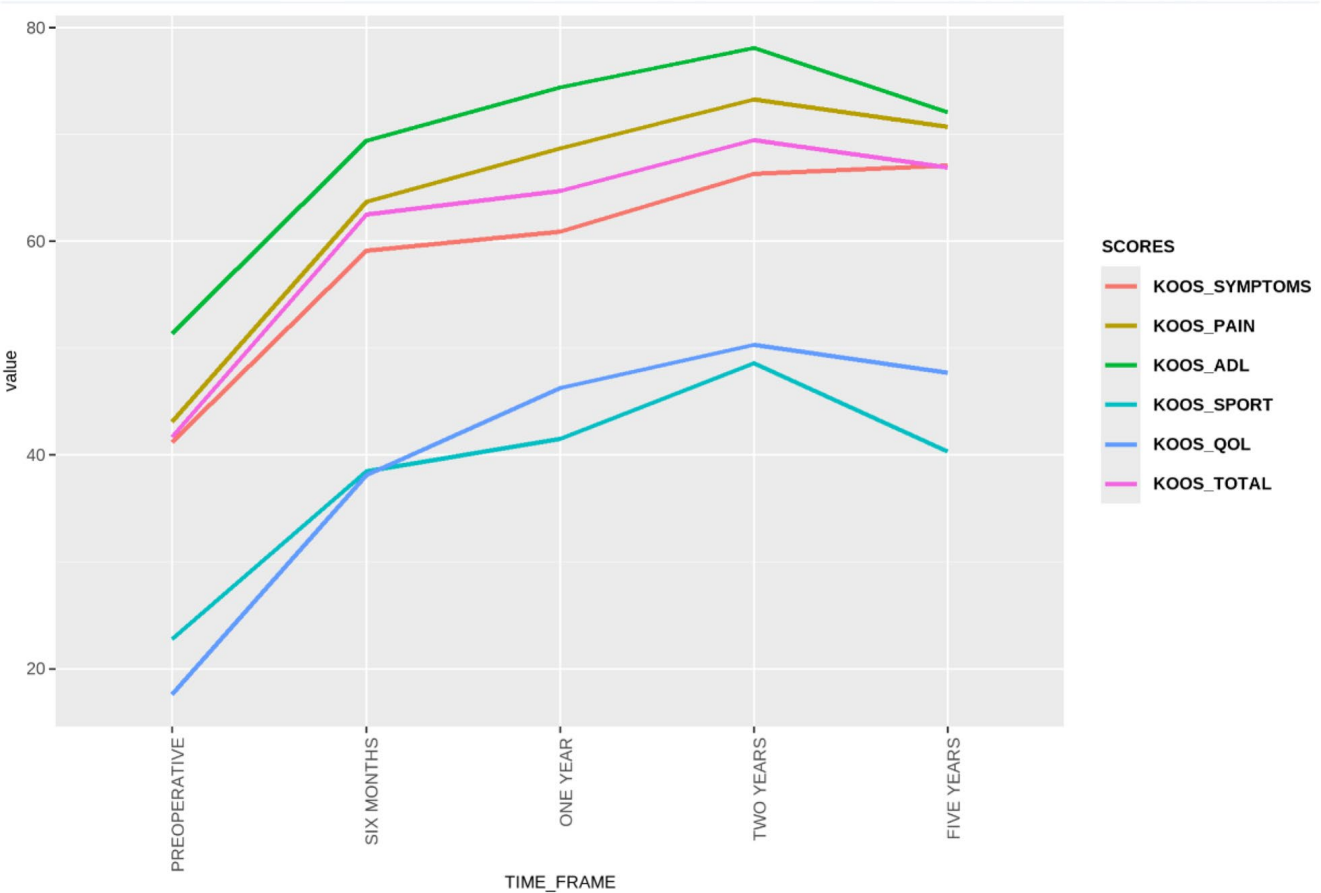
KOOS recorded preoperatively and with a serial progression over a 5 year period

**Fig. 7:**
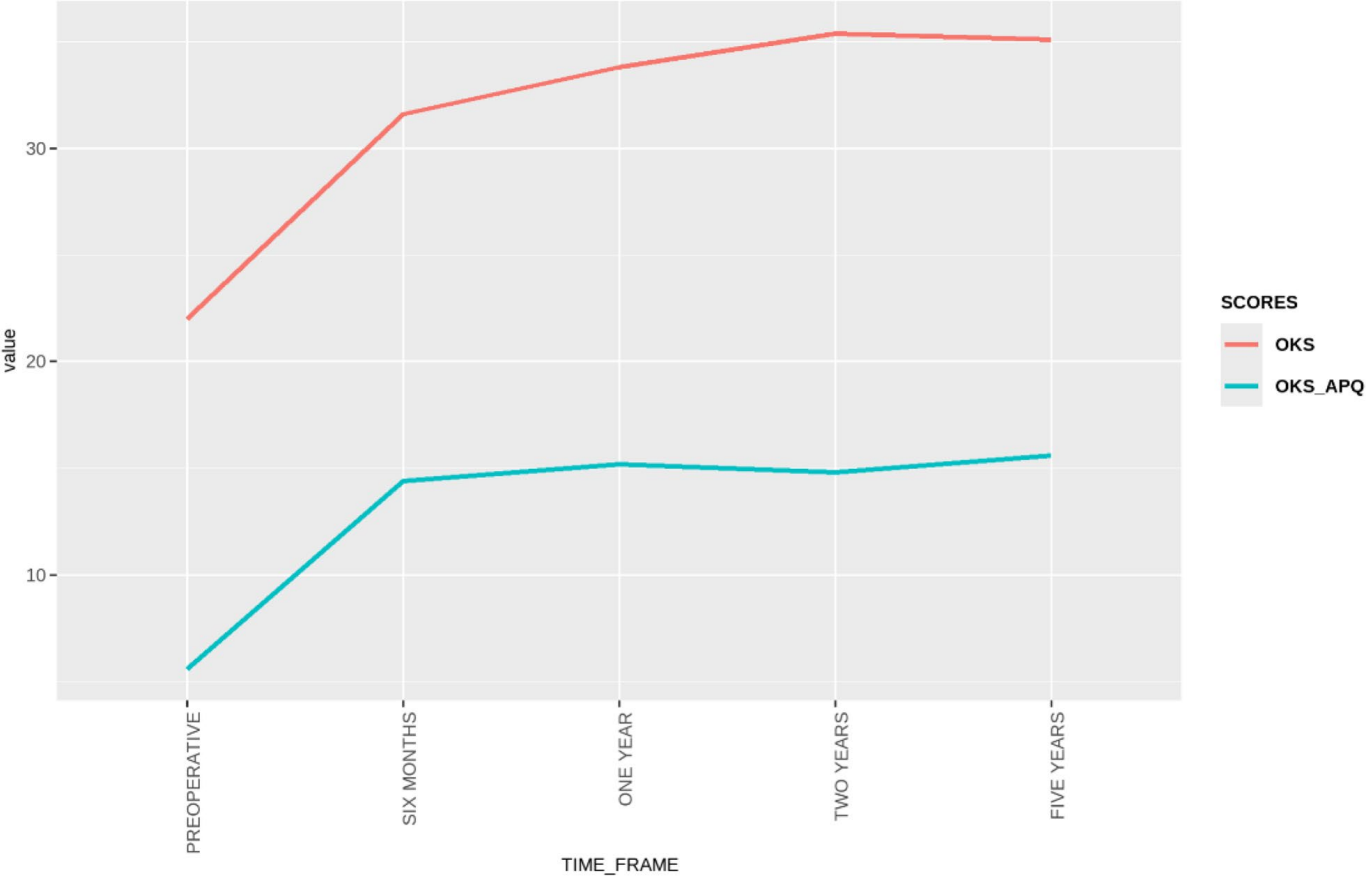
Oxford Knee Scores (OKS) and OKS Activity and Participation Questionnaire (OKS-APQ) recorded preoperatively and in a serial progression over a 5-year period

The linear mixed effects model analysis demonstrated significant improvements in all PROMs over all time points (all *p* < 0.001). The most significant improvements were demonstrated at 2 years postoperatively (Table 5). Neither preoperative BMI (*β* = −0.23, *p* = 0.2) nor preoperative mTFA (*β* = 0.31, *p* = 0.2) were found to be significant predictors of PROM scores. EQ5D analysis is presented in Table 6. Line graphs of all score progressions are presented in Figs. 6, 7, and8.

**Table 5:** Preoperative and regular postoperative recording of KOOS subscales scores, and a variety of PROMs (OKS, OKS-APQ, VAS Health, and VAS pain) over 5 years

	Preoperative	Postoperative 6 months	Postoperative 1 year	Postoperative 2 years	Postoperative 5 years	Delta values (2 years – preoperative)
KOOS symptoms	41.2 ± 18.4 [37.7, 44.8]	59.1 ± 18.9 [55.1, 63.1]	60.9 ± 17.4 [57.5, 64.4]	66.3 ± 18.9 [62.1, 70.6]	67.1 ± 25.0 [59.2, 75.1]	25.1 ± 19.8 [19.7, 28.6]
KOOS pain	43.1 ± 18.3 [39.5, 46.6]	63.7 ± 21.8 [59.1, 68.2]	68.7 ± 19.8 [64.8, 72.7]	73.3 ± 19.3 [69.0, 77.6]	70.7 ± 24.4 [63.0, 78.5]	30.2 ± 21.4 [21.9, 31.5]
KOOS ADL	51.4 ± 23.5 [46.9, 56.0]	69.4 ± 21.2 [64.9, 73.8]	74.4 ± 20.9 [70.2, 78.5]	78.1 ± 20.2 [73.6, 82.7]	72.1 ± 28.0 [63.2, 81.0]	25.8 ± 21.9 [16.8, 26.6]
KOOS sports	22.8 ± 21.0 [18.6, 27.0]	38.5 ± 24.8 [33.2, 43.8]	41.5 ± 29.1 [35.7, 47.3]	48.6 ± 30.3 [41.7, 55.6]	40.3 ± 33.7 [29.5, 51.0]	25.8 ± 31.3 [17.5, 31.9]
KOOS QoL	17.6 ± 15.3 [14.5, 20.6]	38.1 ± 24.0 [33.1, 43.2]	46.3 ± 28.1 [40.7, 51.9]	50.3 ± 27.1 [44.1, 56.6]	47.7 ± 31.5 [37.7, 57.8]	32.7 ± 27.9 [23.1, 35.6]
KOOS total	41.7 ± 18.3 [38.2, 45.2]	62.5 ± 18.6 [58.6, 66.4]	64.7 ± 19.4 [60.9, 68.5]	69.5 ± 19.4 [65.1, 74.0]	66.9 ± 25.0 [59.0, 74.9]	27.8 ± 21.2 [20.2, 29.8]
OKS	22 ± 9.6 [20.1, 23.8]	31.6 ± 10.7 [29.5, 33.8]	33.8 ± 10.9 [31.7, 35.9]	35.4 ± 10.2 [33.1, 37.7]	35.1 ± 11.4 [31.5, 38.7]	13.4 ± 9.9 [8.6, 13.0]
OKS-APQ	5.6 ± 7.1 [4.2, 7.0]	14.4 ± 10.7 [12.2, 16.6]	15.2 ± 11.0 [13.0, 17.4]	14.8 ± 10.7 [12.4, 17.3]	15.6 ± 11.7 [11.9, 19.3]	9.2 ± 10.6 [6.4, 11.1]
VAS health	70.9 ± 23.5 [66.3, 75.5]	75.3 ± 16.7 [71.9, 78.6]	79.8 ± 15.5 [76.7, 82.9]	77 ± 18.0 [72.9, 81.1]	74.8 ± 24.3 [66.8, 82.7]	6.1 ± 23.8 [3.1, 13.9]
WOMAC	43.1 ± 19.4 [38.5, 47.7]	24 ± 17.7 [18.9, 29.1]	26.8 ± 20.2 [21.4, 32.2]	26.1 ± 27.8 [16.8, 35.5]	27.7 ± 20.5 [17.0, 38.4]	−17 ± 30.8 [−32.2, −15.7]
VAS pain	60.2 ± 26.2 [55.0, 65.4]	35.7 ± 24.4 [30.7, 40.8]	31.8 ± 23.5 [27.1, 36.5]	26.7 ± 24.4 [21.1, 32.3]	31.5 ± 29.0 [22.3, 40.7]	−33.5 ± 33.2 [−36.7, −21.8]

*KOOS* Knee Injury and Osteoarthritis Outcome Scores, *ADL* activities of daily living, *QoL* quality of life, *OKS* Oxford Knee Score, *OKS-APQ* Oxford Knee Score—Activity and Participation Questionnaire, *VAS* visual analog scale, *WOMAC* Western Ontario and McMaster University Score

**Table 6:** EQ-5D analysis showing changes in health state **(in percentages)** following DLO in different dimensions at 6 months, 2 years, and 5 years according to the Paretian Classification of Health Change (PCHC) taking into account those with no problems

EQ-5D analysis (PCHC)	EQ-5D dimensions														
	Mobility			Self-care			Usual activities			Pain and discomfort			Anxiety and depression		
	6 M	2 Y	5 Y	6 M	2 Y	5 Y	6 M	2 Y	5 Y	6 M	2 Y	5 Y	6 M	2 Y	5 Y
No change (%)	45	29.4	20	0	9.1	0	42.1	14.7	20	47.6	38.9	30	0	18.8	0
Improve (%)	50	55.9	70	50	27.3	50	36.8	64.7	60	52.4	52.8	65	62.5	43.8	42.9
Worsen (%)	5	14.7	10	50	63.6	50	21.1	20.6	20	0	8.3	5	37.5	37.5	57.1
Total with problems (%)	95.2	94.4	100	19	30.6	10	90.5	94.4	100	100	100	100	38.1	44.4	35
No problems (%)	4.8	5.6	0	81	69.4	90	9.5	5.6	0	0	0	0	61.9	55.6	65

*6 M* 6 months post-DLO, *2 Y* 2 years post-DLO, *5 Y* 5 years post-DLO

**Fig. 8:**
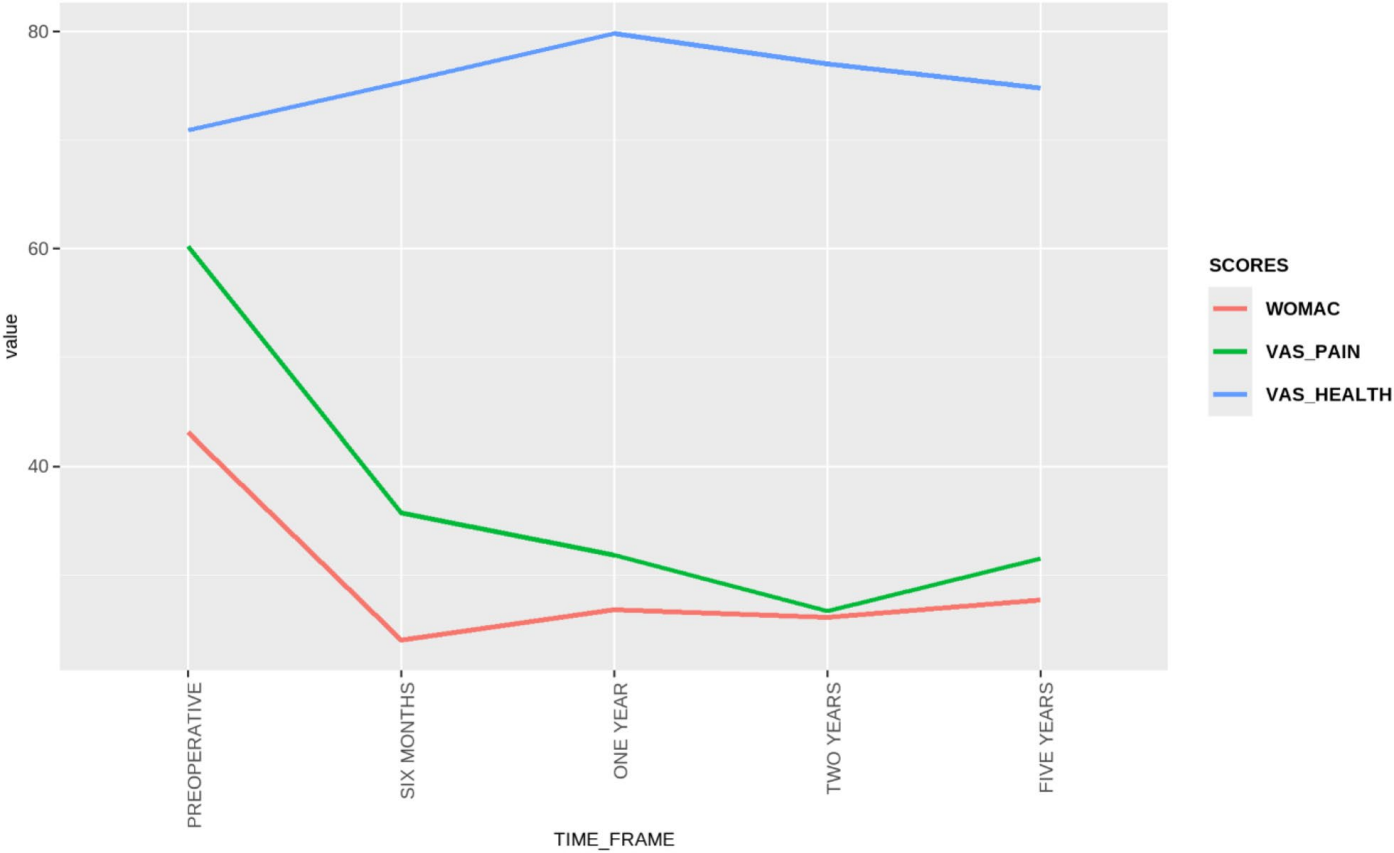
Western Ontario and McMaster University (WOMAC), visual analog scales (VAS) for health and pain scores recorded preoperatively and in a serial progression over a 5-year period

### Complications

The overall complication rate was 8.6% (*n* = 5). The most common reported complication was superficial wound infection 3.4% (*n* = 2), which was managed with oral antibiotics. Details of the recorded complications, the number of cases and their management are recorded in Table 7.

**Table 7:** Complications and treatment of DLO series for varus knees and the recorded number (percentage) of patients for each complication

Complications	Number (%)	Treatment
Failure	**1 (1.7%)**	
Correction loss (DFO)	1	Osteotomy revision
Issues with osteotomy site union	**1 (1.7%)**	
Delayed union (DFO)	1	Revision of compression screw
Infection	**2 (3.4%)**	
Superficial wound infection (DFO)		Antibiotics
Neurovascular	**1 (1.7%)**	
Popliteal artery transection—CPN neuropraxia (HTO)	1	Popliteal artery repair with great saphenous vein interposition graft and Fasciotomy

Bold indicates percentage with regard to the whole series of 58 cases

*CPN* common peroneal nerve

### Revision cases and conversion arthroplasty cases

A total of 5 cases (8.6%) underwent either revision osteotomy or conversion to arthroplasty following the primary DLO procedure. Two cases (3.4%) underwent revision osteotomy using a different plate, with or without bone grafting, at 19 and 24 months postoperatively. Three cases (5.2%) underwent conversion to total knee arthroplasty (TKA) at an average of 5.9 ± 3.1 years postoperatively. No cases were converted to unicompartmental knee arthroplasty (UKA).

**Fig. 9:**
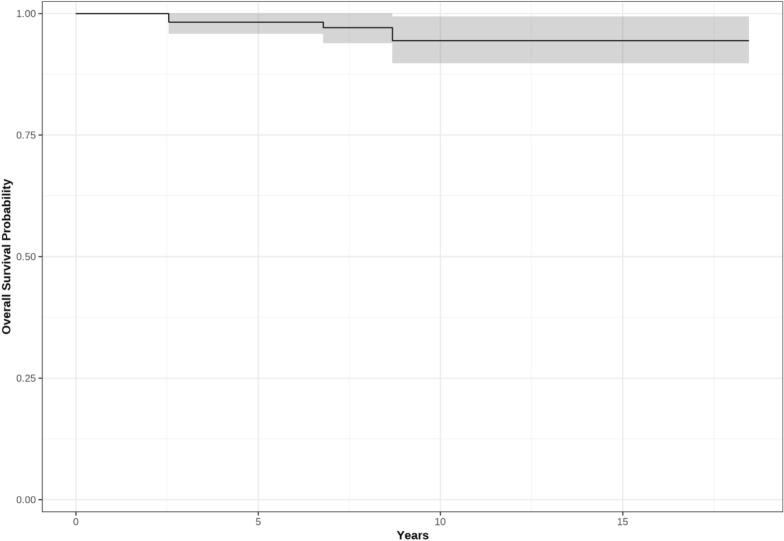
Kaplan–Meier survival curve of DLO for varus knees (black line), with 95% confidence intervals (gray shading)

Survival analysis revealed that the 8-year survival rate of DLO performed for knees with varus malalignment was 97.1% [95% CI 93.9%, 100%]. The survivorship fell to 94.4% [95% CI 89.7%, 99.4%] at 10 years. A Kaplan–Meier Survival analysis curve is shown in Fig. 9.

A Cox proportional hazards model demonstrated that increasing age was significantly associated with a higher hazard of reduced survivorship (HR = 1.27; 95% CI 1.03, 1.55; *p* = 0.02), Similarly, a higher preoperative BMI was also a significant predictor of reduced survivorship (HR = 1.18; 95% CI 1.01, 1.37; *p* = 0.034).

## Discussion

The most important findings in the present study (a single-center series of 58 valgization DLO for varus knees), are the improved clinical outcomes, a low complication rate of 8.6%, and a 94.4% ten-year survivorship. Knee DLO was first reported by Benjamin in 1969 [39]. Historically, there have been reports indicating poor results and high complications following DLO; however, the cases were a mix of rheumatoid arthritis and osteoarthritis [40–42]. Nevertheless, reports from the modern era have challenged the bad reputation of this procedure, especially with better patient selection and improved fixation modalities [17, 21, 25, 29, 43].

In the presented series, the cases had severe varus malalignment with a preoperative mTFA of −12 ± 3.9°, valgization DLO restored neutral limb alignment to postoperative mTFA of −0.4 ± 3.4°, and the postoperative MPTA and mLDFA were normalized to 90 ± 2.5° and 86.7 ± 2.5°, respectively. In addition, there was a significant improvement in all PROMs, represented by a variety of scoring systems, which was noted at all the follow-up points up to 5 years postoperatively. Similarly, Schröter et al. [29], in a series of 37 varus malaligned knees with preoperative mTFA of −11 ± 3°, demonstrated that valgization DLO could restore neutral alignment with postoperative mTFA of 0 ± 2°, without excessive joint line obliquity. In their series, the postoperative MPTA and mLDFA were normalized to 89.2 ± 2° and 87 ± 2°, respectively, and there was significant improvement in PROMs [29]. These findings, in line with previous studies, highlight the efficacy of this procedure both radiologically and clinically [20–22, 24, 25, 29].

The results show that the mean mechanical outcome is a marginal under-correction from a neutral mTFA with 0.4° of persisting varus. However, normalization of the mechanical axis from the severe varus starting position (mean −12.7° of varus) adequately redistributed the load to the unaffected compartment, as demonstrated by the resultant clinical improvement in patient symptoms and significant improvement in all PROMS. This is consistent with the prime principle of coronal plane osteotomy of unloading an overloaded compartment [36, 44].

Terauchi et al. [45] investigated the recurrence of varus deformity following HTO and reported an association between distal femur varus deformity and increased horizontal joint obliquity. It was noted that excessive joint obliquity, following HTO, prevents load redistribution to the lateral compartment and contributes to the recurrence of varus deformity [45]. In the presented series, the preoperative mean MPTA was 84.3 ± 3.2° and the preoperative mean mLDFA was 91.6 ± 3.4°, which indicated bifocal varus deformities. Therefore, valgization DLO was indicated to correct the deformity at both sites and avoid an excessive joint line obliquity [2]. Previous studies of valgization DLO have reported similar indications [19].

Abs et al. [33], in a comparaison of DLO versus OWHTO for patients with bifocal (femur and tibia) varus deformity, demonstrated significant superiority for the DLO group in all the radiological indices, especially in maintaining the joint line obliquity (JLO). In addition, superior UCLA (University of California,Los Angeles) scores and higher satisfaction were reported in DLO compared with OWHTO; however, there was no significant difference in KOOS or return to work or sports between both groups. Furthermore, hinge fractures were significantly fewer in the DLO group compared with the OWHTO group [33]. Similarly, Akamatsu et al. [19] demonstrated superiority for DLO to maintain JLO compared with OWHTO, and higher reported Lysholm scores in DLO compared with OWHTO in cases with bifocal varus deformity. These studies further emphasize the conclusion of the present study, with DLO as a more rewarding surgical option for cases with bifocal varus deformity compared with a single level OWHTO.

There is scarce literature reporting on the complications following valgization DLO. Babis et al. [21] reported only one case (3.4%) that collapsed into valgus due to insufficient femoral fixation. However, the femoral fixation in their series was conducted with either a dynamic condylar plate or a blade plate [21]. In the modern era with angular stable fixations, this is unlikely to be experienced unless the osteotomy is complicated with a missed hinge fracture that is not addressed properly. Grasso et al. [22] reported complications in 9.1% of cases (*n* = 2) including postoperative hematoma and deep wound infection.

In the presented series, the overall complication rate was 8.6% (*n* = 5). This is even lower than the overall complication rate for the largest published series of MOWHTO at 10.3% [14]. Nevertheless, a serious complication in one case (1.7%) was reported, which involved popliteal artery transection and common peroneal nerve (CPN) neuropraxia. Therefore, the relatively low complication rate should not underestimate the complexity of this procedure, which necessitates precise planning and careful execution to achieve the desirable outcomes with the lowest possible complications. It must also be noted that these surgeries were undertaken in a high-volume center by experts in the field.

In the presented series, a high survivorship of 97.1% at 8 years remained high at 94.4% at 10 years. Conversion to arthroplasty was reported in three cases (5.2%) at an average of 5.9 ± 3.1 years. This matches what was reported by Babis et al. [21], who evaluated a series of 29 valgization DLOs for varus knees, where a cumulative rate of survival at 8.3 years was reported to be 96% [21]. They reported only one case (3.4%) that required conversion to TKA at 4.1 years [21].These findings underscore the high effectiveness of this procedure in delaying the need for an arthroplasty procedure in the long term.

A correlation between excessive JLO and inferior outcomes following HTO is still controversial. Akamatsu et al. [19] compared the clinical, radiographic, and arthroscopic outcomes of DLO versus HTO in two groups of 34 knees, after adjustment for demographics and hip knee angle. A significant postoperative increase in JLO was noted in the HTO group versus the DLO group, from 1.4° to 6.3° versus 1° to 1.3°, respectively [19]. However, there was no difference between the two groups in either regeneration of the cartilage of the medial tibial plateau or degeneration of the cartilage of the lateral tibial plateau [19]. In another study, Akamatsu et al. [46] compared clinical, radiographic, and arthroscopic outcomes between two groups (43 knees each): one group with a postoperative MPTA ≤ 95° and another group with a postoperative MPTA of > 95°, after adjustment for the preoperative MPTA. A lower KOOS sports and recreational function subscale score was reported in the group with MPTA of > 95° at 2 years postoperatively, but no difference in articular cartilage appearance between both groups at 1-year follow-up [46].

Similarly, Rosso et al. [47] investigated the prognostic factors related to the clinical outcomes following opening wedge HTO at 10 years of follow-up and concluded that no correlation exists between excessive joint line obliquity or MPTA ≥ 95° and the clinical outcomes at 10 years of follow-up [47]. Xie et al. [48], in a series of 463 knees that had lateral closed wedge HTO for OA and varus malalignment, demonstrated that increased postoperative JLO (MPTA ≥ 95°) does not influence HTO survivorship at 5, 10, and 15 years [48].

On the contrary, Horita et al. [49], in a series of 52 knees which underwent MOWHTO, demonstrated lower Japanese knee outcome measures at the last follow-up in cases with increased postoperative JLO. However, in their series, the mean age was 61.6 ± 9 years, which could have contributed to the inferior clinical outcomes due to the progression of OA [49].

There are some limitations of the presented series including the retrospective nature of the study, albeit with prospectively collected outcome data. Another limitation is the lack of a comparative group with patients who had a single level osteotomy; however, this has been addressed by presenting a comparative discussion on the existing literature with HTO. The study reports on short-term to midterm outcomes up to 5 years; however, 10-year outcomes are being collected and will be reported in the literature. Finally, there is heterogeneity in the fixation devices and void-filling techniques; however, all devices were angular stable fixation devices.

## Conclusions

In this single-center series evaluating patients with varus knees and bifocal deformities, valgization double-level knee osteotomy (DLO) demonstrated favorable clinical outcomes, accompanied by a low complication rate of 8.6% and a 10-year survivorship of 94.4%. Radiographic findings from available imaging data were positive, although long-term imaging was not consistently obtained.

## Data Availability

Data is available upon request with an approved permission from Basingstoke and North Hampshire Hospital.

## Abbreviations

BMI: Body mass index
DFO: Distal femoral osteotomy
DLO: Double level osteotomy
HTO: High tibial osteotomy
JLCA: Joint line convergence angle
KL: Kellgren–Lawrence
KOOS: Knee Injury and Osteoarthritis Outcome Score
LCW: Lateral closing wedge
LDTA: Lateral distal tibial angle
LL: Lower limb length
LSRs: Long leg standing radiographs
MOW: Medial opening wedge
MOWHTO: Medial opening wedge high tibial osteotomy
MPTA: Medial proximal tibial angle
mLPFA: Mechanical lateral proximal femoral angle
mLDFA: Mechanical lateral distal femoral angle
mTFA: Mechanical tibiofemoral angle
OA: Osteoarthritis
OKS: Oxford Knee Score
OKS-APQ: Oxford Knee Score—Activity and Participation Questionnaire
PCHC: Paretian Classification of Health Change
PROMs: Patient-reported outcome measures
SCQ: Self-administered comorbidity questionnaire
TKA: Total knee arthroplasty
VAS: Visual analog scale
WOMAC: Western Ontario and McMaster University Score
